# Determination of object position, vortex shedding frequency and flow velocity using artificial lateral line canals

**DOI:** 10.3762/bjnano.2.32

**Published:** 2011-06-06

**Authors:** Adrian Klein, Horst Bleckmann

**Affiliations:** 1 Institute for Zoology, University of Bonn, Poppelsdorfer Schloss, 53115 Bonn, Germany

**Keywords:** artificial lateral line, biomimetics, flow sensor, mechanoreception, optical sensor

## Abstract

The lateral line system of fish consists of superficial neuromasts, and neuromasts embedded in lateral line canals. Lateral line neuromasts allow fish to sense both minute water motions and pressure gradients, thereby enabling them to detect predators and prey or to recognize and discriminate stationary objects while passing them. With the aid of the lateral line, fish can also sense vortices caused by an upstream object or by undulatory swimming movements of fish. We show here that artificial lateral line canals equipped with optical flow sensors can be used to detect the water motions generated by a stationary vibrating sphere, the vortices caused by an upstream cylinder or the water (air) movements caused by a passing object. The hydrodynamic information retrieved from optical flow sensors can be used to calculate bulk flow velocity and thus the size of the cylinder that shed the vortices. Even a bilateral sensor platform equipped with only one artificial lateral line canal on each side is sufficient to determine the position of an upstream cylinder.

## Introduction

Nature has invented a stunning diversity of sensory systems whose small size and high sensitivity is so far unmatched by man-made devices. Flow sensors based on hairs are located on the skin and are found in crustaceans [1], as well as in spiders and insects [2]. These sensors enable insects and spiders to perceive air displacements down to flow amplitudes of 30 μm/s [3]. Flow sensors are also found in fish and aquatic amphibians and are called lateral line neuromasts. With neuromasts some fish can detect water surface waves with a displacement amplitude of only 0.01 μm [4]. Most lateral line neuromasts are located on the skin (superficial neuromasts or SN), but some are located in subepidermal canals (canal neuromasts or CN).

A lateral line neuromast consists of up to several hundred or even thousand mechanosensitive hair cells, enveloped in a gelatinous cupula. Lateral line hair cells are morphologically polarized, i.e., they have a bundle of stereovilli at their apical surface that grows longer from one edge to the other. Within the sensory epithelium of a neuromast, lateral line hair cells are oriented into two opposing directions that define the most sensitive axis of the neuromast [5]. Although the design of the lateral line can be quite different in different fish species [6], most fish have several hundred (sometimes up to several thousand) SNs distributed over their head, body and tail fin [7].

Fish use their lateral line for prey detection, predator avoidance, schooling, intraspecific communication, rheotaxis and station holding [8–9]. In addition, with the lateral line system fish can form a spatio-temporal image of nearby objects based on their hydrodynamic signature [10].

Lateral line afferents not only respond to the sinusoidal water motions generated by a vibrating sphere [11–12] but also to more natural stimuli, such as single vortices [13] or vortex streets caused by a cylinder exposed to running water [14]. The direction of water motions inside a vortex as well as the vortex shedding frequency of a cylinder can be determined from the responses of primary lateral line afferents [13–14]. Lateral line nerve fibers also alter their discharge rate if an object passes the fish laterally. In this case the discharge pattern reflects both the position (including distance) of the object and the direction of object motion [15].

The sensory hairs of crustaceans, insects and spiders and the lateral line system of fish have inspired engineers to develop artificial air [16] and water flow sensors [17–19] based on microelectromechanical system (MEMS) technology. With individual sensors, mimicking SN or sensory hair function, the air or water motions caused by a vibrating sphere can be detected [16], and, if arrays of multiple sensors are used, a vibrating sphere can even be localized [17].

Nature alters the response properties of biological flow sensors by altering their shape and size [20]. In case of the lateral line, the canals also determine the response properties of the flow sensors. In general, lateral line canals shield CNs from DC flow [12]. In most fish species the course of the lateral line canals is clearly visible to the naked eye since lateral line canals open to the environment through a series of pores. In teleosts, CNs are located halfway between neighbouring canal pores. Drag forces, resulting from fluid motions induced inside the canal by pressure fluctuation outside of the canal, stimulate CNs. In general, CNs respond (up to a frequency of about 150 Hz) roughly in proportion to the pressure gradient between neighbouring pores and thus to the external water acceleration [21]. SNs respond (up to about 80 Hz) roughly in proportion to the water velocity [22].

There have been several attempts to design an engineering equivalent to the sensory hairs of insects and spiders [16,23] and to the superficial neuromast system of fish. However, an engineering equivalent of the fish lateral line canal system did not previously exist, and therefore we have built artificial lateral line canals (ALLCs) and equipped them with artificial neuromasts (ANs). Unlike the artificial neuromasts built by other researchers, our neuromasts use optical signals to measure water or air motions. With these optical flow sensors we measured water (air) motions inside artificial canals and thus we were able to quantify outside pressure gradients. The performance of the ALLCs was assessed by exposing them, not only to the pressure gradients caused by a stationary vibrating sphere, but also to the vortical flows caused by a cylinder placed upstream in running water. We show that ALLCs can even be used to determine the position and size of an upstream cylinder and to detect and localize a moving object.

## Results

### Experiment I

**Figure 1:**
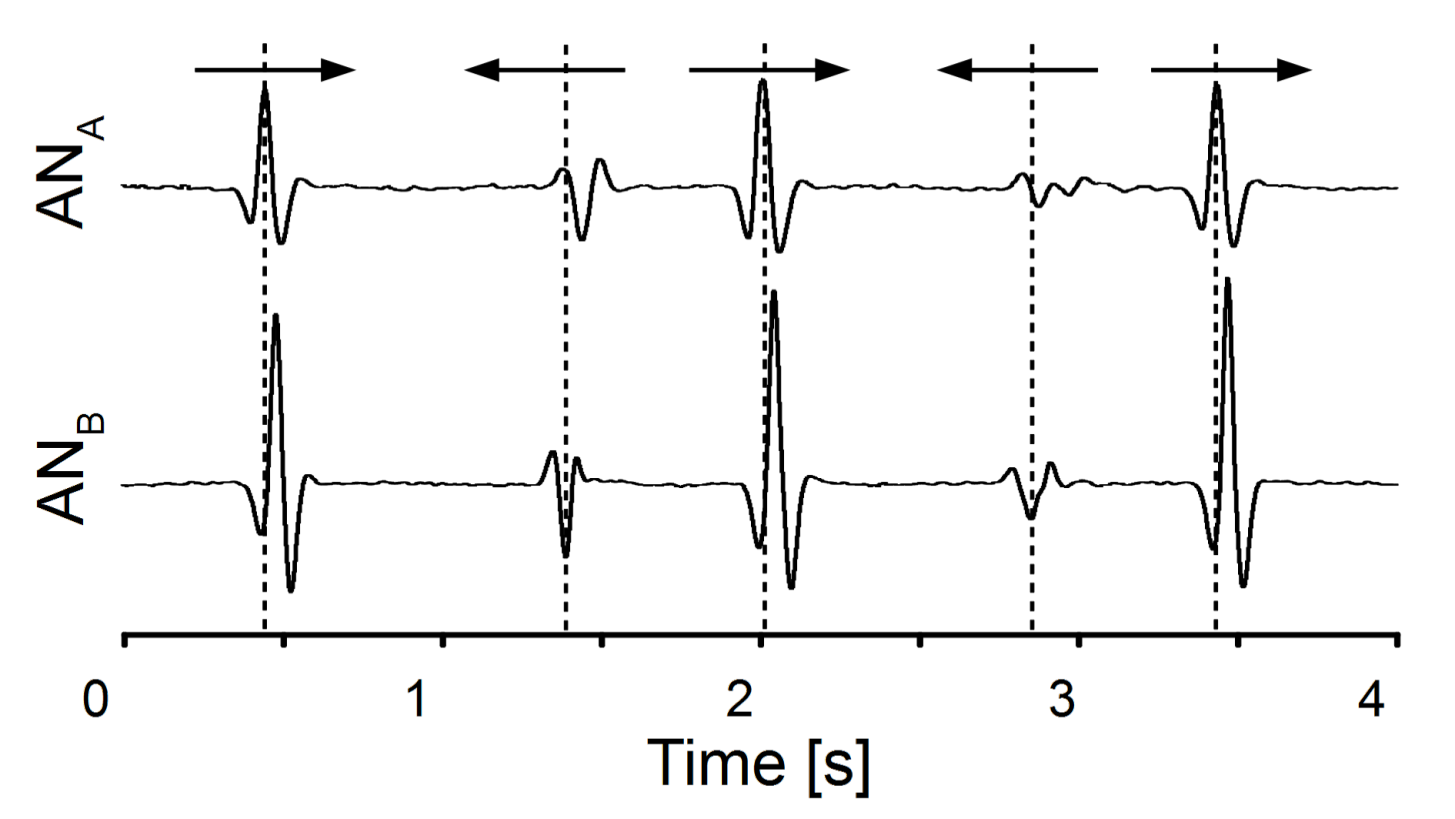
Responses of sensor platform I to a human finger that moved alongside the ALLC with a velocity of about 20 cm/s. Arrows indicate direction of finger motion. Responses of AN_A_ (upper graph) and AN_B_ (lower graph) are shown. Note that the responses were inverted if the direction of finger motion was reversed.

In experiment I we investigated whether ALLCs can be used to detect object movements in air. To stimulate the ALLC, a human finger was moved alongside the ALLC at a distance of about 2 cm. The finger movement caused responses that had a ‘Mexican hat shape’, i.e., a decrease in voltage amplitude was followed by an increase and another decrease (Figure 1). If the direction of finger movement was reversed, the shape of the response also reversed. A canal with only two neuromasts was sufficient to determine the velocity of finger movement. If the finger first passed AN one (AN_A_), the response of AN two (AN_B_) was delayed with respect to the response of AN_A_. Thus artificial lateral lines (ALLs) equipped with only two neuromasts can not only be used to detect moving objects but can also determine object velocity and roughly the direction of object motion.

### Experiment II

**Figure 2:**
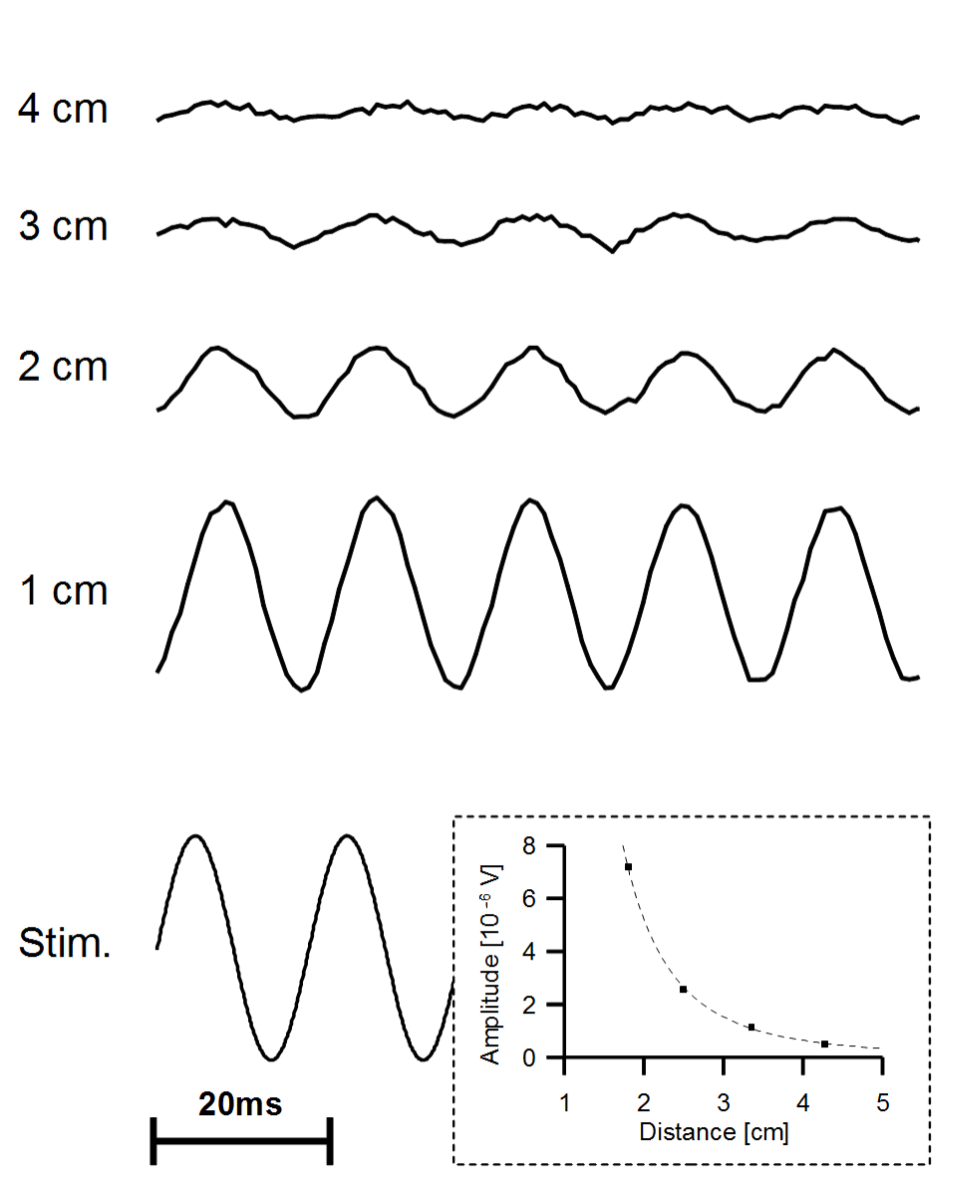
A typical AN response to a dipole flow field. The sinusoidal voltage used to drive the mini-shaker (bottom left) and the voltage output of the AN exposed to the vibrating sphere (top 4 traces) is shown. Distances between the ALLC and the vibrating sphere were 1, 2, 3 and 4 cm. The voltage output of the AN is plotted on the same scale. Inset: Voltage output of the AN as a function of sphere to pore distance. The fitting function (dashed line) is f(x) = 42.66 x^−3.03^. This indicates a third order decrease of sensor output with increasing sphere distance.

To examine the performance of ALLCs in water, a vibrating sphere (diameter 1 cm, p–p displacement amplitude 237 μm, vibration frequency 50 Hz, duration of vibration 200 ms) was placed adjacent to the ALLC. Within the range of distances tested (1 to 4 cm), the frequency of the voltage output of the AN equalled the frequency of sphere vibration (Figure 2). As expected [22], the output voltage of the AN decreased approximately with the third power of the sphere distance (see inset Figure 2). With respect to sphere movement, the voltage output of the AN was phase shifted by 90°.

### Experiment IIIa

**Figure 3:**
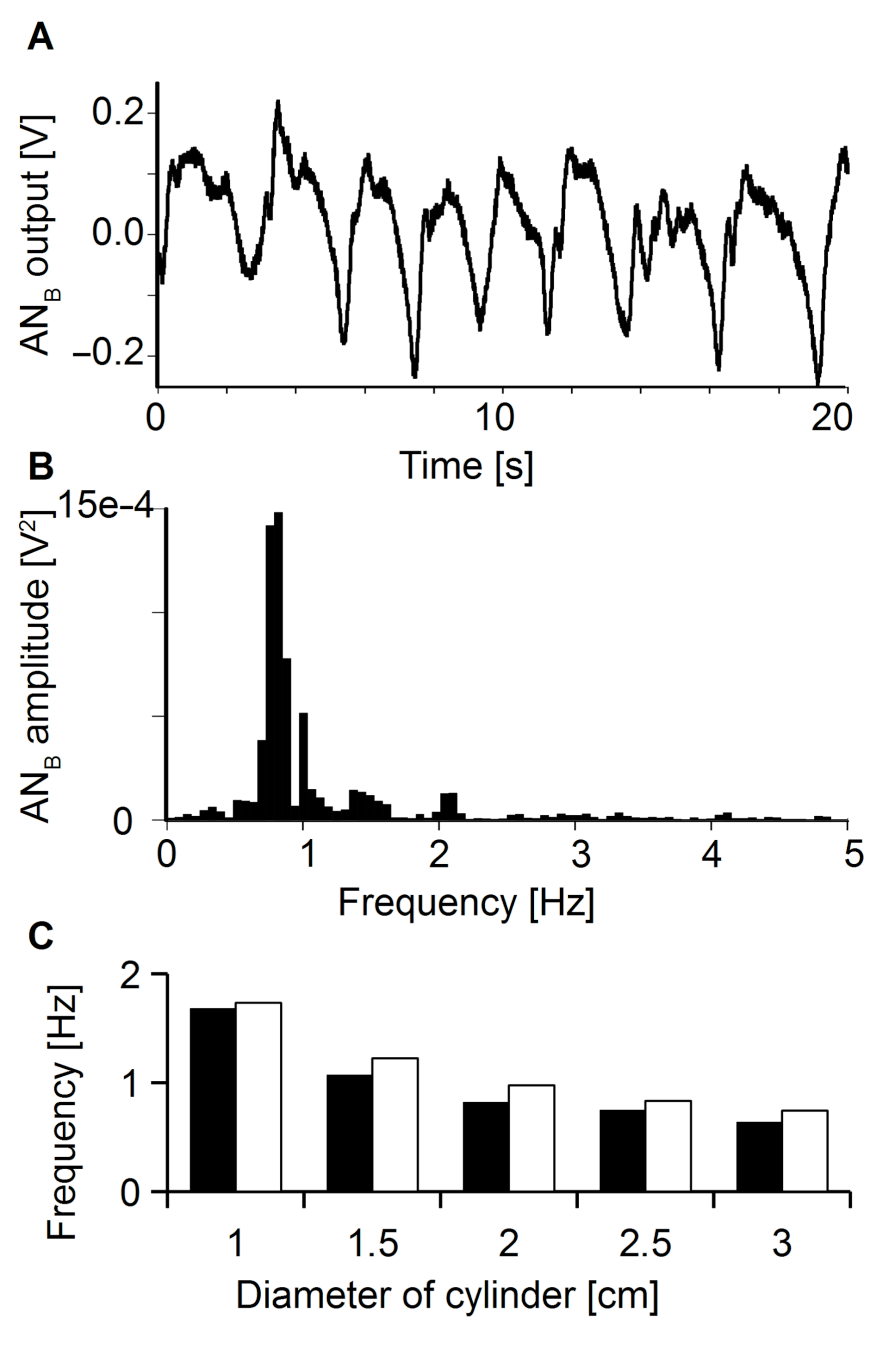
The response of an artificial CN exposed to the vortices shed from a stationary cylinder (diameter 2 cm) (A) and frequency spectrum of the response (B). Peak frequency (0.82 Hz) was similar to the calculated vortex shedding frequency (0.98 Hz). (C) Measured (black bars) and calculated (white bars) vortex shedding frequencies for cylinders of different diameters.

This experiment tested whether ALLCs can be used to detect an upstream cylinder exposed to running water. ANs responded to such a cylinder with an oscillating voltage output (Figure 3A). The Fourier spectra of these oscillations had a peak frequency (Figure 3B) close to the calculated (see material and methods) vortex shedding frequency (VSF) of the cylinder. When the diameter of the cylinder was altered, this oscillation peak shifted in the predicted (Equation 1) direction, i.e., with increasing cylinder diameter the VSF decreased (Figure 3C).

### Experiment IIIb

**Figure 4:**
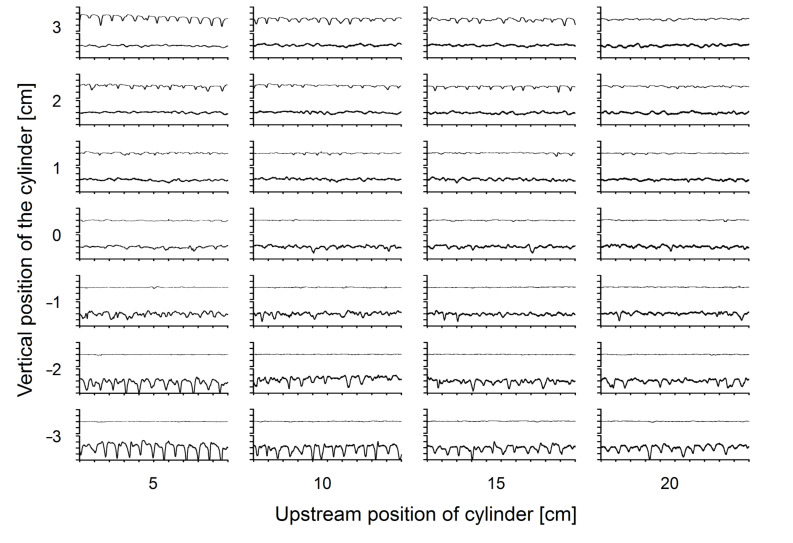
Responses of AN_A_ (upper line in each graph) and AN_B_ (lower line in each graph) (for AN_A_ and AN_B_ see Figure 6A) to the vortices caused by a cylinder (diameter 2 cm) placed at various positions upstream to the ALL. Note that response amplitudes decreased with increasing distance of the cylinder and that the responses were more pronounced for the AN that was ipsilateral to the cylinder.

**Figure 5:**
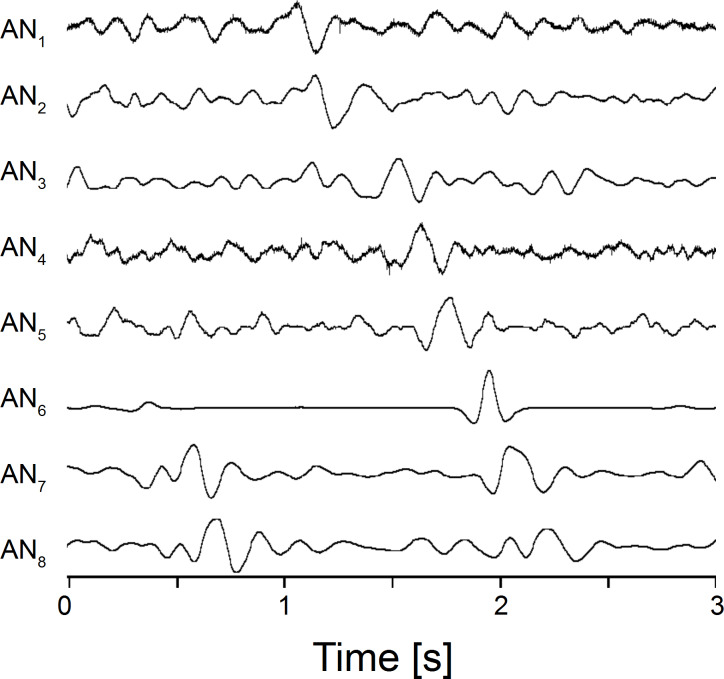
Responses of AN_1_ to AN_8_ (cf. Figure 6B) to bulk water flow. Note that the most prominent peak visible in the response of AN_1_ is systematically delayed from AN_1_ to AN_8_. The curves are not to scale due to the manual fabrication of each AN.

In this experiment we tested whether ALLs can also be used to localize an upstream cylinder. When the distance between the ALLC and the cylinder was increased, the voltage output of the AN decreased, but the upstream cylinder could still be detected from a distance of 20 cm. When the cylinder was moved perpendicular to the direction of bulk water flow, the responses recorded from the ipsilateral ALLC were larger than the responses recorded from the contralateral ALLC (cf. Figure 4). In general, moving the cylinder to the left or right systematically changed the responses of the two ALLCs (Figure 4). Thus by comparing the voltage output of the two ALLCs (Figure 5) we could approximately determine the lateral position of the cylinder.

**Figure 6:**
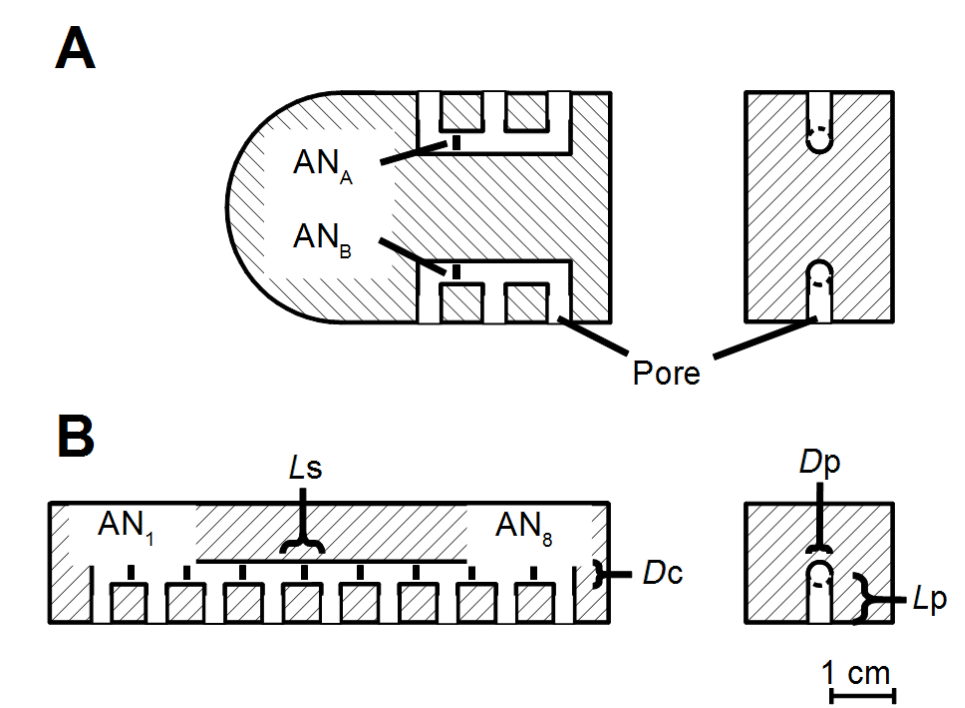
Horizontal (left) and vertical (right) cross sections of platforms III (A) and IV (B) that were used for the present study. Black squares indicate the positions of the ANs inside artificial lateral line canals. Platform III housed two and platform IV housed eight ANs. The length of pores (*L*p) was 1 cm, the inter pore distance (*L*s) was 1 cm and the diameter of the canal (*D*c) as well as the pore diameter (*D*p) was 3 mm in both devices.

### Determination of bulk flow velocity

**Figure 7:**
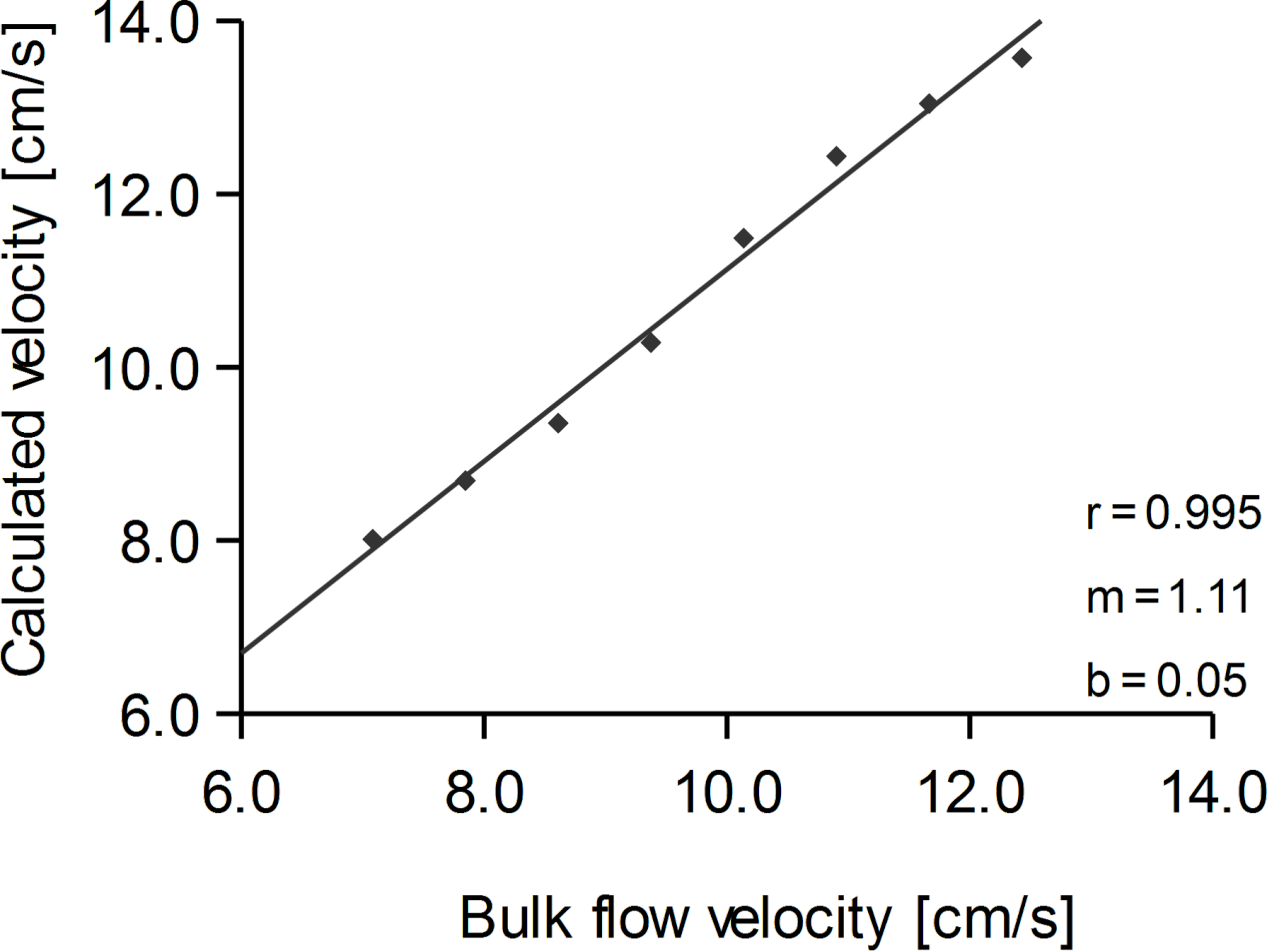
Velocity calculated with data obtained from AN_1_ to AN_8_ as function of bulk flow velocity. Note that the fitting curve intersects close to the origin (b = 0.05). Its slope (m) is larger than 1. This could be due to vibrations or a crosstalk between adjacent ANs.

To determine bulk flow velocity, the responses of all eight ANs of sensor platform IV (cf. Figure 6B) were compared. If the ALLC of sensor platform IV was exposed to running water, the responses of all ANs showed characteristic peaks (e.g., Figure 7). Any particular distinctive part of the voltage output (e.g., a peak) was first generated by the specific AN that was located at the most upstream position. In ANs located more downstream, this part occurred with delays that were proportional to the downstream distance of the respective neuromast. We calculated bulk flow velocity by cross correlating the output data of neighbouring ANs (see also [24]) and dividing the pore distance by the correlation delay. Mean bulk flow velocity was determined using the calculated bulk flow velocities obtained from 7 adjacent neuromast pairs.

## Discussion

There have only been a few attempts to design an engineering equivalent to the sensory hairs of insects and spiders [16,23]. These sensors were used for air flow sensing and for aerial acoustic perception [16,18]. Researchers also have built artificial lateral line neuromasts. Campenhausen et al. [19] built an artificial SN that consisted of a needle that connected a plastic blade (the artificial cupula) with a piece of paper that partly intercepted a light beam. In standing water, this system was able to sense vertically oriented metal bars while passing. Yung et al. [25] built a monolithically integrated array of microfabricated hot wire anemometers. The hot wires had a length of 400 µm, an elevation of 600 µm and a spacing of 1 mm. This matches the dimensions of many fish lateral lines. The anemometer system exhibited a sensitivity of about 200 μm/s flow velocity, a bandwidth of about 1 kHz, and, due to its small dimensions, a reduced interference with the flow field and with the neighbouring anemometers. Unfortunately, hot wire anemometers cannot discern flow direction. As a result, they only provide a rectified reading of oscillatory flow fields. Nevertheless, arrays of hot wire anemometers were successfully used to determine the location of a moving dipole source up to a distance equalling the length of the sensor array, to access the signature of a wake caused by an upstream object and to determine the general direction to the location of that object [25]. Recently, Yan et al. [17] built an ALL that consisted of superficial microfabricated ANs wrapped around a cylinder. Each of the 15 ANs consisted of a horizontal cantilever with a vertical hair (length 500 μm) attached at the distal end. At the fixed end of the cantilever was a piezoresistor. Flow that impinged upon the vertical hair created a bending torque that acted on the cantilever to induce stress concentration at the site of the piezoresistor. The induced change of resistance was converted to a voltage and thus could be used to infer the local flow velocity. Unlike sensors based on hot-wire anemometry, these sensors were directional like lateral line neuromasts, i.e., along their dominant axis they responded to water flow in both directions with equal sensitivity but with opposite polarity. The detection limit for water flow was 100 μm/s, a value comparable to the fluid velocity detection thresholds of SNs (38 to 60 μm/s [26–27]).

Some researchers [28–30] have also studied the filter properties of ALLCs. To do so they measured optically particle displacements inside ALLCs in relation to particle displacements in the outside medium. According to these studies, particle displacements inside ALLCs are related linearly to those in the medium. Below 80 Hz, the ratio of particle displacement inside the canal to particle displacement in the medium roughly followed the velocity in the medium. Water displacements in the ALLC were proportional to the component of the external velocity parallel to the canal. If a cylinder is placed near to an ALLC, an externally exposed flow induces complicated flow patterns inside the canal that depended on the position of the cylinder relative to the ALLC [30]. There was little mechanical coupling between neighbouring parts of the canal.

The present study is the first to measure the performance of ALLCs equipped with optical ANs. This has enabled us to illustrate the potential of optical ANs and of biomimetic ALLCs. In general, ALLCs can be used to measure and quantify air and water motions, but due to the different Reynolds numbers, larger flow speeds are needed in air compared to water to obtain a signal. In comparison with a pure SN-system, a canal system has several advantages: 1) Sensors situated in canals are not exposed to the external environment, thus they cause minimal interference to the external flow field; 2) the sensors are protected from the external environment and thus are less prone to physical damage; 3) the sensitivity, frequency response and dynamic amplitude range of ALLCs can easily be altered by changing canal morphology (e.g., the number, size and placement of canal pores, canal diameter, and canal compartmentalization) [31] and 4) by building artificial canals that have tubuli [6], the mechanical filter properties of ALLCs can be further altered in a predictable manner [32].

With the aid of ALLCs equipped with optical flow sensors, we were able to detect sinusoidal water motions. The responses were phase shifted by about 90°, i.e., as expected [22] the ALLC acted as a differentiator. According to the flow field equations [21], at a distance of 4 cm from a vibrating sphere (1 cm diameter, 50 Hz, 237 µm p–p sphere displacement) the tangential flow amplitude is about 4 µm/s (about 12.7 nm displacement). Thus a sinusoidal signal with this amplitude could be detected with our ALLCs. Besides a stationary vibrating sphere, the ALLCs responded to linear object motions and to the vortices shed by a cylinder. A stationary sensor platform equipped with two ALLCs (one on each side) was sufficient to determine the approximate upstream position of a cylinder. And a canal equipped with at least two ANs could determine bulk flow velocity. If the variables bulk flow velocity and vortex-shedding frequency are known, then the size of the cylinder can also be calculated (cf. Equation 2).

According to a recent study an artificial cupula made out of hydrogel can improve the performance of artificial sensory hairs (ANs) by about two orders of magnitude. Minimal thresholds were as low as 2.5 μm/s [33]. Thus, one should be able to increase further the sensitivity of ALLCs by attaching artificial cupulae to the artificial CNs.

Overall, our study shows that ALLCs equipped with optical flow sensors are effective for the detection and quantification of aerial or aquatic stimuli. ALLCs could potentially provide unprecedented sensing and control functions to underwater vehicles and platforms. If so they may be useful for guiding autonomous vehicles (in both air and water), tracking and identifying wake generators and for the measurement of air and water movements in pipes and canals. To improve the performance of ALLCs, one important step will be to reduce the size of the optical ANs using MEMS technology. This is currently being done in cooperation with the center for advanced studies (caesar) in Bonn. Smaller sensors will allow us to build smaller canal systems and thus to miniaturize the sensor platforms. Finally, the development of ALLs will facilitate fundamental studies on the fish lateral line.

## Experimental

### Sensor platforms

Half canals were milled inside opaque PVC plates. Two mirror inverted PVC plates were screwed together thereby forming a sensor platform that contained ALLCs. Four sensor platforms were built. Type I had a single linear canal that consisted of 3 segments and 4 canal pores (length *L*s = 12.5 mm, pore length *L*p = 5 mm, canal diameter *D*c = 4 mm, pore diameter *D*p = 4 mm, for definitions see Figure 6). Type II had a single linear canal that consisted of only one segment (*L*s = 30 mm, *L*p = 10 mm, *D*p = 4 mm, *D*c = 4 mm) with 2 canal pores. Type III had two linear canals (one on each side; cf. Figure 1A), each of which consisted of 2 segments and 3 pores (*L*c = 10 mm, *L*p = 5 mm, *D*c = 3 mm, *D*p = 3 mm) and type IV had one linear canal that consisted of 8 segments and 9 canal pores (*L*c = 10 mm, *L*p = 5 mm, *D*c = 3 mm, *D*p = 3 mm; cf. Figure 1B).

### Artificial canal neuromasts

**Figure 8:**
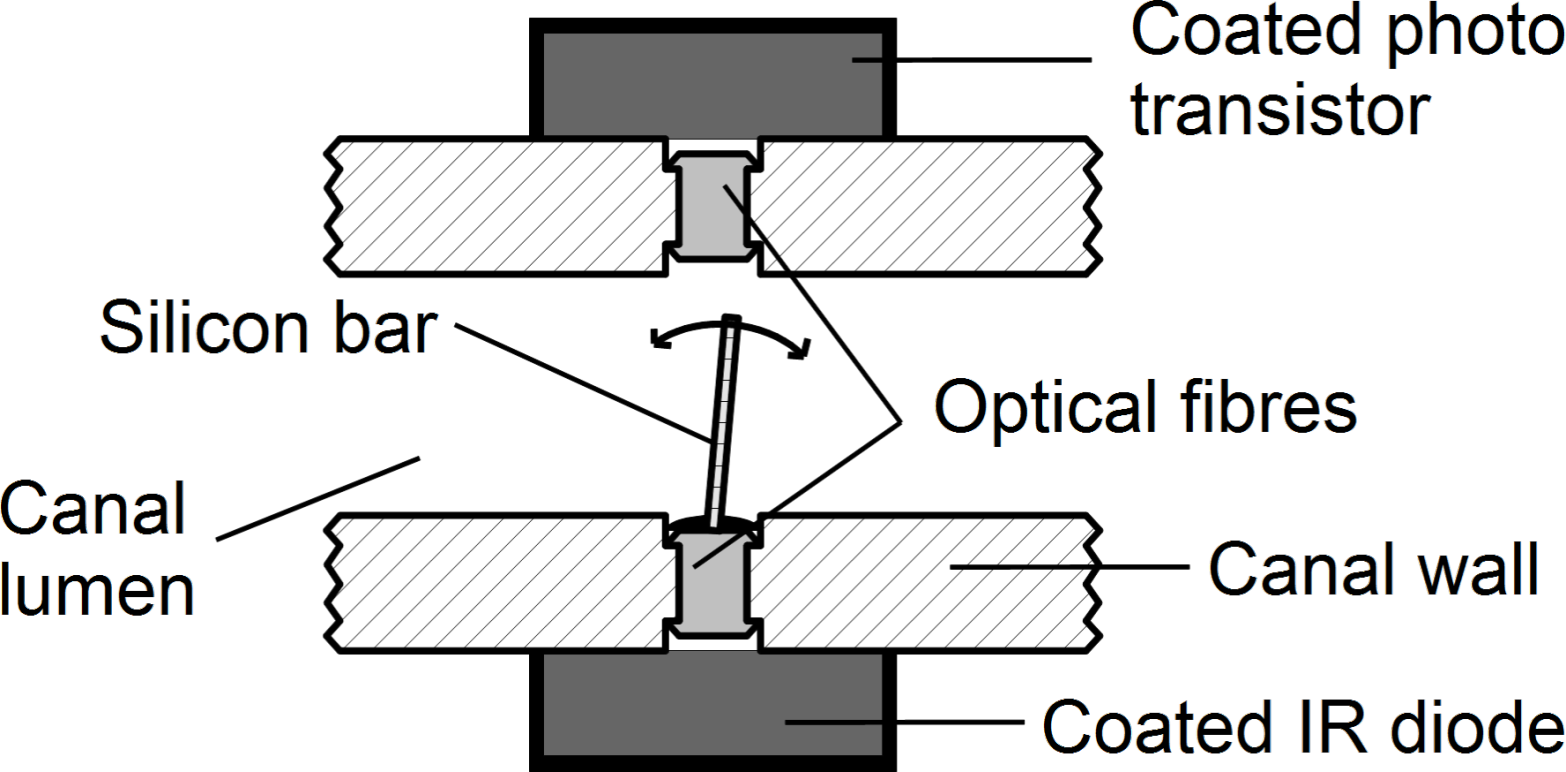
Scheme of an artificial CN. The drawing is not to scale.

ALLCs were equipped with artificial canal neuromasts (artificial CNs). To build artificial CNs, transparent silicone bars (Wacker Silicones, Elastosil RT 60) were fabricated (length < *D*c, width 0.4–1.0 mm) and installed inside each ALLC segment (cf. Figure 8). The density of the silicone bars was comparable to the density of water to ensure that only the motion of the fluid affected the bending of the bar. For the detection of canal fluid motion, one end of each silicone bar was illuminated with an infrared light emitting diode (LED, SFH420). Light, leaving the opposite end of the silicone bar, illuminated an optical fiber that was connected to a SMD phototransistor (PT15) (cf. Figure 8) and the output was amplified (transistor BC547). A potentiometer (100 kΩ) was placed between the emitter of the transistor and earth. The transistor output was fed into an AD converter (Power 1401, sampling rate 1 kHz) and stored on a computer (Optiplex 745, Dell). Pressure gradients between canal pores caused water (or air) flow inside the ALLCs that in turn deflected the silicone bars. In the absence of a stimulus, the silicone bars were not perfectly vertically oriented (0°) but instead were slightly deflected to one side (at about 10°, cf. Figure 8). The degree of deflection modulated, i.e., increased (flow in one direction) or reduced (flow in the opposite direction), the amount of light that reached the SMD phototransistor. Thus, the artificial CNs were directionally sensitive.

### Test of sensor performance

To test the performance of the sensor platforms, four experiments were performed:

#### Experiment I

Sensor platform I (not shown) was used. A human finger moving in air alongside the platform (the ALLC) served as a stimulus source.

#### Experiment II

Hydrodynamic stimuli were generated with a vibrating sphere (diameter 1 cm, 50 Hz, 237 µm peak to peak displacement) that was attached to a stainless steel rod (length 10 cm, diameter 1.6 mm). The rod was mounted on a mini-shaker (Ling Dynamic System LTD, model V 101) that rested on a sliding bar assembly allowing for manual adjustment of the sphere distance to the type II sensor platform (also not shown). The sphere was positioned at the height of the ALLC midway between two canal pores. The distance between the surface of the sphere and the sensor platform was 1, 2, 3 or 4 cm. The vibration direction of the sphere was parallel to the long axis of the ALLC.

#### Experiment III

The sensor platform was submerged in a flow tank that had a cross section of 10 × 15 cm^2^ and a water depth of 12 cm. It contained a flow collimator upstream of the working section holding the sensor platform. Flow (7.9 cm/s) was produced by a propeller (Schottelantrieb Aeronaut 100) coupled to a d.c. motor (Conrad Electronic) that was driven by a power supply (Voltcraft DIGI 35, Conrad Electronic). The propeller was suspended from a holder on the side of the tank opposite to the section that housed the sensor platform. For experiment III sensor platform III (cf. Figure 6B) was used.

#### Experiment IIIa

The sensor platform was positioned horizontally in the middle of the working section of the flow tank. In this way both canals of the platform were exposed to bulk water flow. To generate vortices, a half-cylinder (diameter 1.5 cm, hereafter referred to as the cylinder; Reynolds number 1200) was placed vertically in the working section of the flow tank. The distance between the cylinder and the upstream edge of the sensor platform was 5, 10, 15 or 20 cm. The lateral distance of the cylinder with respect to the midline of the sensor platform was −3, −2, −1, 0, +1, +2 or +3 cm.

#### Experiment IIIb

One side of sensor platform III was attached to the wall of the flow tank, i.e., only one canal was exposed to bulk water flow. Cylinders with a diameter of 1, 2 or 3 cm were placed 10 cm upstream from the sensor platform. The lateral distance between the cylinders and the midline of the sensor platform was 2.5 cm.

#### Experiment IV

Sensor platform IV was used (cf. Figure 6B). A cylinder (diameter 2 cm) was placed 10 cm upstream from the sensor platform. The in-plane curved head (radius 1.5 cm) of the platform served to minimize any fluctuations that may have been caused by the upstream edge of the sensor platform. Bulk flow velocity was varied between 3.3 and 12.4 cm/s.

#### The generation of defined vortex streets

Over a certain range of Reynolds numbers (typically above 140), a cylinder placed in running water will generate a Kármán vortex street. The frequency of vortex detachment (*f*) is a function of the Strouhal number S_t_ (a dimensionless index), the diameter *d* of the cylinder and the actual flow velocity (*v*) [34]:

[1]
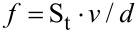


The Strouhal number for the cylinder depends on the Reynolds number; however, it remains at an almost constant value of 0.2 for Re > 2000 [33]. To account for flow constrictions near the cylinder due to blocking effects, the VSF was calculated using the actual flow velocity (*v*) in the region of the cylinder, according to a method typically used for vortex flow meters [35–36]:

[2]
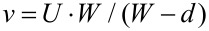


where (*U*) is the nominal flow velocity (7.9 cm·s^−1^ in our experiments) and *W* the width of the flow tank (10 cm). For the experiments a Reynolds number between 800 and 2400, based on the diameter of the cylinder, was chosen.

Bulk flow velocity was measured with particle image velocimetry (experiment 1, 2, and 3, cf. [37]) or with an impeller (P670).
